# Developing ovine mammary terminal duct lobular units have a dynamic mucosal and stromal immune microenvironment

**DOI:** 10.1038/s42003-021-02502-6

**Published:** 2021-08-20

**Authors:** Dorottya Nagy, Clare M. C. Gillis, Katie Davies, Abigail L. Fowden, Paul Rees, John W. Wills, Katherine Hughes

**Affiliations:** 1 Department of Veterinary Medicine, University of Cambridge, Cambridge, UK; 2 Department of Physiology, Development, and Neuroscience, University of Cambridge, Cambridge, UK; 3 College of Engineering, Swansea University, Fabian Way, Crymlyn Burrows, Swansea, UK; 4 Broad Institute of MIT and Harvard, Cambridge, MA USA; 5 Present Address: Equine Clinic, Department of Companion Animals and Equids, Faculty of Veterinary Medicine, University of Liège, Liège, Belgium

**Keywords:** Innate immunity, Lymphoid tissues, Image processing, Machine learning, Developmental biology

## Abstract

The human breast and ovine mammary gland undergo striking levels of postnatal development, leading to formation of terminal duct lobular units (TDLUs). Here we interrogate aspects of sheep TDLU growth as a model of breast development and to increase understanding of ovine mammogenesis. The distributions of epithelial nuclear Ki67 positivity differ significantly between younger and older lambs. Ki67 expression is polarised to the leading edge of the developing TDLUs. Intraepithelial ductal macrophages exhibit periodicity and considerably increased density in lambs approaching puberty. Stromal macrophages are more abundant centrally than peripherally. Intraepithelial T lymphocytes are more numerous in older lambs. Stromal hotspots of Ki67 expression colocalize with immune cell aggregates that exhibit distinct organisation consistent with tertiary lymphoid structures. The lamb mammary gland thus exhibits a dynamic mucosal and stromal immune microenvironment and constitutes a valuable model system that provides new insights into postnatal breast development.

## Abstract (web summary)

Nagy et al. describe the histomorphogenesis of ovine mammary development in pre- and peri-pubertal sheep as a candidate model for human breast development. They investigate the locale and abundance of several immune cell types at different ages, highlighting this as a valuable model system that can provide new insights into postnatal breast development.

## Introduction

The mammary gland undergoes a dramatic degree of postnatal growth, developing from a rudimentary branched structure at birth to an arborizing bilayered ductal network in the nulliparous adult.

Mammary development has been widely studied in rodents. Macrophages are key players in the direction of murine mammary ductal growth^1^ and there is increasing recognition of a spectrum of mammary macrophage subsets^2^. Mammary macrophages may be derived from the foetal liver and yolk sac and additionally infiltrate postnatally^3^. Depletion experiments have demonstrated the dependence of mammary postnatal development on macrophages^4^ and that alveolar bud formation and ductal epithelial proliferation are reduced in their absence^5^. Stat5 is activated in mammary macrophages during development, and mice with macrophages that have conditional deletion of Stat5 exhibit perturbed development^6^. Cells expressing MHCII are closely associated with murine mammary ducts^7,8^, and macrophages envelop the pubertal terminal end buds^9^. The atypical chemokine receptor ACKR2, which scavenges CC-chemokines, has been implicated in macrophage recruitment during mammary development^10,11^. Intriguingly, macrophage depletion of virgin mice also influences the mammary stromal extracellular matrix composition, highlighting the importance of macrophages in both the epithelial and stromal compartments^12^.

CD4^+^ and CD8^+^ lymphocytes have also been identified in the murine mammary gland^13,14^. As T-cell receptor alpha deficient mice exhibit enhanced ductal outgrowths, it is postulated that T lymphocytes may act in a negative regulatory manner^13^. Similarly, lymphocytes are present in the human breast^15,16^ although little is known about their developmental role.

An understanding of postnatal pre-pregnancy breast development in humans is critical to the interrogation of the pathogenesis of breast diseases^17^. Whilst mouse models of mammary development are highly tractable and extremely valuable, they have inherent limitations and caution has been recommended in the extrapolation of results of murine developmental studies directly to humans^18^. Potentially pertinent given the complex interactions between cellular compartments, mammary epithelial cells in the breast are surrounded by fibrous connective tissue whereas the murine mammary stroma is adipose-rich^19^. By contrast, the ruminant mammary gland exhibits a strikingly similar micro-anatomical arrangement of terminal duct lobular units (TDLUs) and fibrous stroma to the human breast^19,20^. We and others have therefore suggested that it represents a valuable adjunctive model of the breast TDLU^21,22^ although further interrogation of the utility of this model is required.

The phase of pre-pubertal development of the bovine mammary gland has been highlighted as a developmental period that impacts future lactational productivity^23,24^ and a number of studies have provided valuable insights into pre-pregnancy development of the bovine udder, including the effect of plane of nutrition on its development^25^. To assess epithelial proliferation in the bovine mammary gland, bromodeoxyuridine (BrdU) incorporation studies have been performed, and these have revealed cell proliferation to be higher in the peripheral parenchymal zone than adjacent to the developing gland cistern^26,27^. In heifers, the mass of mammary parenchyma is reduced in ovariectomized animals^28^. Assessments of immune cell distribution have demonstrated that stromal macrophages are more abundant within 100–150 μm of the bovine mammary epithelium, and that macrophage frequency is negatively affected by ovariectomy^29^. The presence of mast cells and eosinophils has also been demonstrated^29^.

Sheep are frequently used as a model species in foetal development studies^30^ and also constitute a globally valuable production animal species. Studies have indicated that pre-pregnancy development of the ovine mammary gland is characterised by periods of allometric growth, suggested to be between 3 and 4 months of age^31^ or 10–15 weeks of age^32^. Interestingly, important differences between ovine and bovine mammary development have been identified. For example, ovine prepuberal allometric mammary growth is unaffected by ovariectomy^33^. Such differences underline the need for further ovine-specific mammary developmental studies. In particular, there is a relative paucity of data on the presence of immune cells during ovine pre-pregnancy mammary development.

Given that studying ovine mammary development will offer new insights relevant to breast development, and that there is a pressing need for species-specific data regarding udder development in the pre-pregnancy ewe, we sought to capitalise on the availability of new technologies to study postnatal mammary development in this species. We hypothesised that the pre-pregnancy period would be punctuated by distinct periods of epithelial proliferation that would not be uniform across the gland. Based on the extensive data suggesting a critical role for immune cells in murine mammary development, we also anticipated that this phase of ovine postnatal development would be characterised by immune cell fluxes. We therefore utilised deep learning image analysis to assess epithelial proliferation by means of Ki67 staining in ovine mammary TDLU development and employed two-dimensional (2D) and deep three-dimensional (3D) imaging approaches to interrogate and quantify the presence of macrophages, lymphocytes and tertiary lymphoid structures within the gland during development.

## Results and discussion

### Mammary epithelial proliferation is considerably greater in younger lambs than in those approaching puberty, with proliferation focused at the leading edge of the advancing TDLUs

**Fig. 1:**
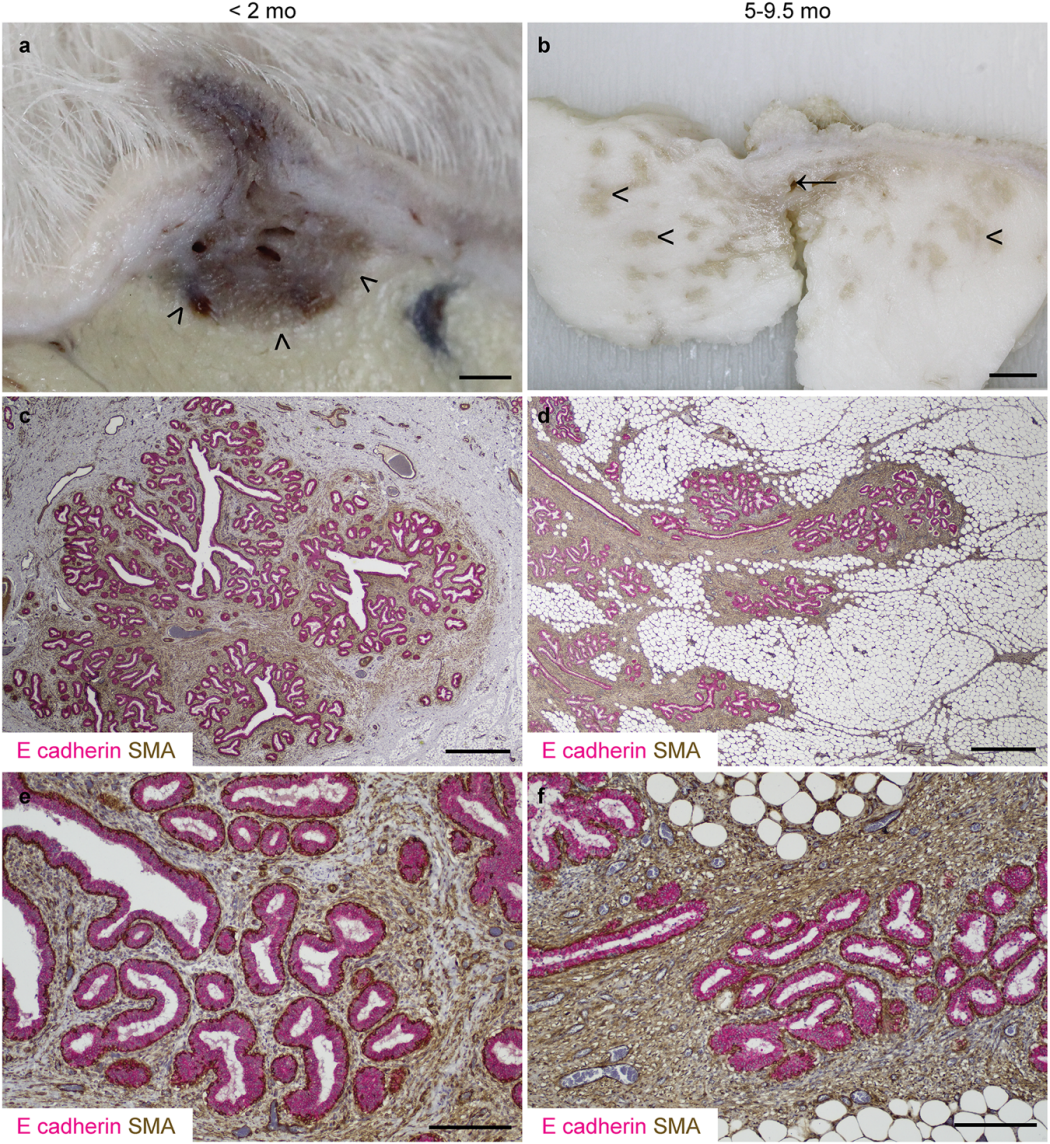
Lamb terminal duct lobular units (TDLUs) advance into the mammary fat pad during postnatal development. **a**, **b** Sub-gross images of fixed mammary tissue. Arrowheads indicate the developing mammary TDLUs infiltrating the mammary fat pad. Arrow indicates rudimentary gland cistern. **c**–**f** Immunohistochemical staining for E-cadherin (magenta) & alpha-smooth muscle actin (SMA; brown). Haematoxylin counterstain. Bar: 1.5 mm (**a**); 5 mm (**b**); 800 μm (**c**, **d**); 200 μm (**e**, **f**). Images are representative of five biological repeats.

Preclinical models of tumourigenesis do not always portray the heterogeneity of human disease^34^, and this limitation may also apply to developmental studies where a relatively homogeneous population of rodents, maintained in controlled conditions, may not recapitulate the diversity of the progression of breast development noted in humans^15^. For this study, we therefore selected a heterogeneous population of pre- and peri-pubertal lambs of differing breeds, maintained in different husbandry systems. This population of lambs exhibit developing TDLUs supported by intra- and interlobular stroma (Fig. 1), very similar to the breast, and in contrast to the murine mammary gland^19^. Similar to the case in heifers, alpha smooth muscle actin is expressed intensely by mammary basal epithelial (myoepithelial) cells and less intensely by cells within the intralobular stroma^35^.

**Fig. 2:**
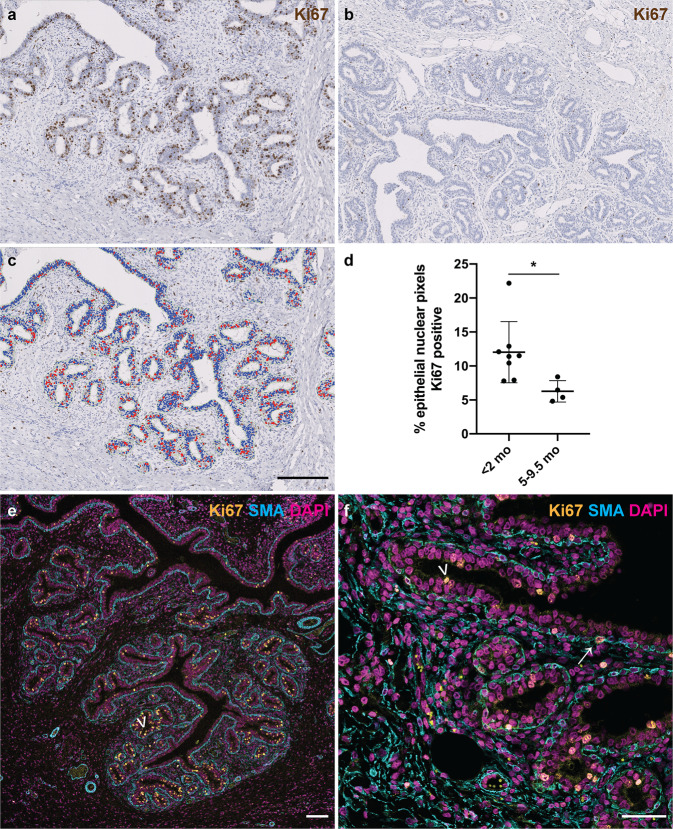
Mammary epithelial proliferation is significantly higher in younger lambs than in those approaching puberty. **a**–**c** IHC for Ki67 in mammary gland from lambs < 2 mo (**a**) and 5–9.5 mo (**b**) and accompanying mask derived using an algorithm detecting intra-epithelial Ki67 positive events (**c**). **d** Scatter plot demonstrating that the distributions of epithelial nuclear Ki67 positivity differ significantly between younger and older lambs. Dots represent individual lambs. Bars represent mean ± standard deviation. * *p* < 0.05. **e**, **f** IF for Ki67 (gold), α-SMA (cyan) and DNA (DAPI; magenta) demonstrating that the majority of Ki67 positive nuclei are in the luminal epithelial layer (arrowheads), with rare Ki67 positive nuclei in myoepithelial cells (arrow). **e** 1 do lamb. **f** 9.5 mo lamb. do, days old; mo, months old. Images are representative of eight (**a**), four (**b**), twelve (**c**) and three (**e**, **f**) biological repeats. All IHC images have haematoxylin counterstain. Scale bar = 200 μm (**a**–**c**); 100 μm (**e**); 50 μm (**f**).

To assess nulliparous ovine mammary growth dynamics, we performed immunohistochemical staining (IHC) for Ki67 to delineate actively cycling cells. The abundance of epithelial nuclear Ki67 positivity differs significantly (*P* = 0.0162) between lambs less than 2 months old compared to peri-pubertal lambs aged 5-9.5 months old, with considerably higher levels of epithelial proliferation observed in younger lambs (Fig. 2a–d). This finding builds upon a historic study using dried fat-free tissue weights to assess mammary growth that suggested that ovine allometric mammary growth occurred at 3–4 months old, prior to puberty. Notably, that analysis was somewhat limited in scope, with only Romney and Romney-cross animals examined and no animals older than 5 months old included in the pre-pregnancy group^31^. Similarly, other authors have demonstrated a period of allometric ovine mammary growth between 10 and 15 weeks of age^32^. In bovines, a period of allometric mammary growth occurring between 2 and 3 months and 9 months of age has been identified^36^.

In the present study, immunofluorescence staining (IF) demonstrates that although the majority of epithelial proliferation is luminal, myoepithelial (basal) cells occasionally express Ki67 (Fig. 2e, f). This highlights similarities with the breast, where sporadic proliferating myoepithelial cells have been noted in normal breast parenchyma of women aged 30–68 years, using samples where biopsies or mass removal has included normal tissue^37^. In our ovine sample set, myoepithelial proliferation appears to be a highly stochastic phenomenon, with incidence of Ki67 positive nuclei ranging between 1 in 4 and 1 in 155 myoepithelial nuclei. However, the data from recent 3D studies^7,9^ would suggest that assessment of myoepithelial proliferation in 2D would likely ‘undersample’ myoepithelial proliferation events and so we do not consider that our 2D data allow robust quantification of myoepithelial proliferation. There has been a relative paucity of focus on myoepithelial proliferation within the developing breast or mammary gland prior to pregnancy. During lactation, myoepithelial cells contract to deform alveoli, facilitating milk release in response to oxytocin stimulation^38^. Our identification of proliferation within the myoepithelial compartment pre-pregnancy suggests that studying basal epithelial replication during this period may provide new insights into udder development, possibly affecting lactation efficiency.

**Fig. 3:**
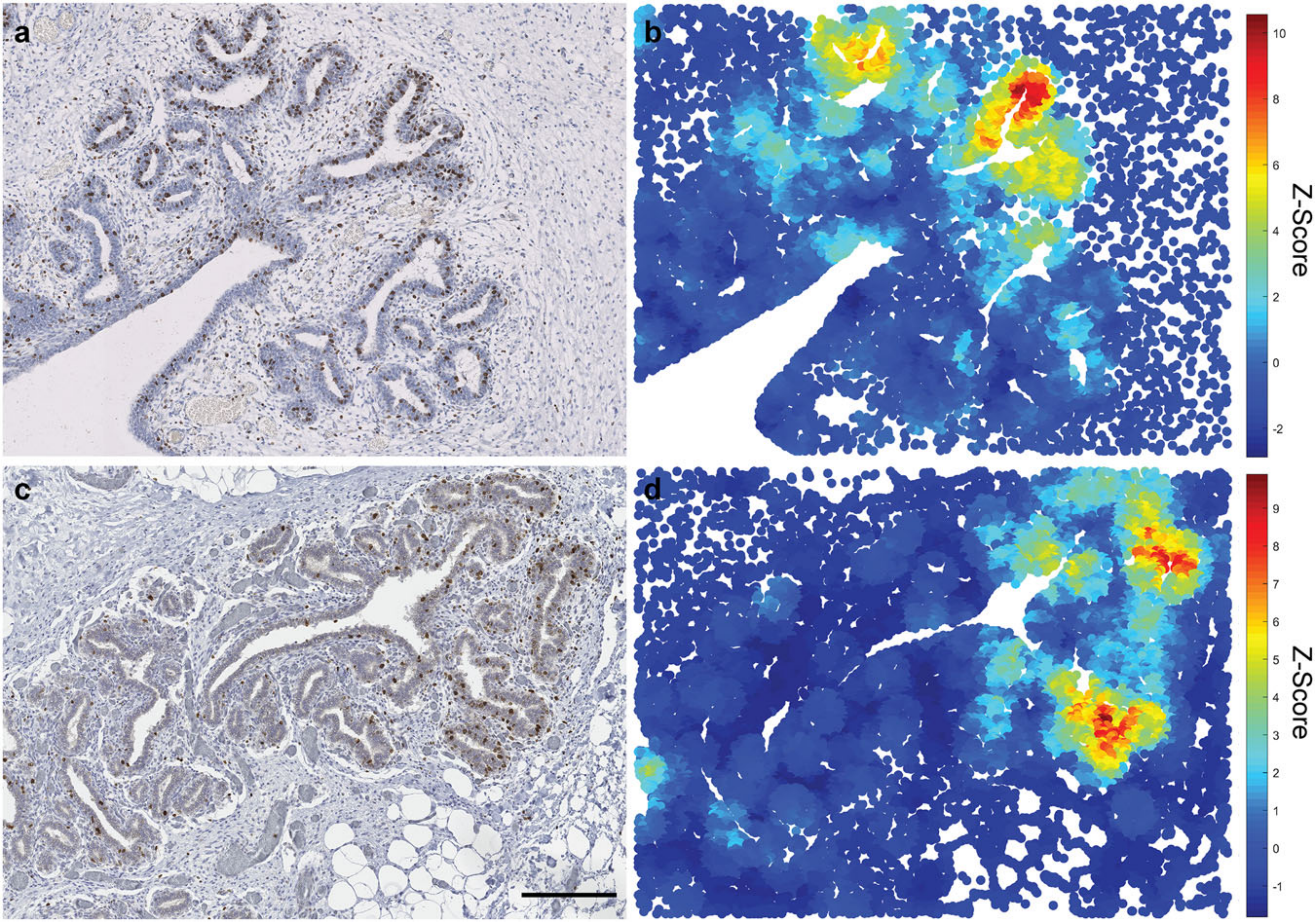
The developing lamb mammary gland exhibits polarity of Ki67 epithelial expression with Ki67 expression focused at the leading edge of the advancing TDLUs. IHC for Ki67 (**a**, **c**) and accompanying Getis-Ord (G-O) statistical analyses (**b**, **d**) demonstrating regions with significant spatial congregation of intraepithelial Ki67^+^ cells (scale (*d*) parameter = 250 px). Mammary gland from lambs < 2 mo (**a**, **b**) and 5-9.5 mo (**c**, **d**); mo, months old. **a**, **c** Haematoxylin counterstain. Scale bar = 200 μm. **b**, **d** Results are representative of three biological repeats.

Having observed that pre-pregnancy ovine mammary epithelial proliferation is not temporally uniform, we wished to interrogate the spatial distribution of Ki67-positive epithelial events. Spatial statistical analyses (Getis-Ord GI*) reveal distinct polarisation of epithelial proliferation towards the advancing tips of the developing TDLUs (Fig. 3a–d), echoing non-quantified description of non-random localisation of Ki67 expression in the infant breast^17^ and similar distributions of BrdU incorporation in bovine mammary epithelium^27^. This finding further underlines the utility of the lamb mammary gland as a model of breast development. Interestingly, although the rat mammary gland exhibits prominent histo-anatomical differences from that of the ruminant, qualitative descriptions of a similar phenomenon of Ki67 polarisation have also been made, with Ki67 positivity focused in the terminal end buds^39^.

### Macrophages exhibit spatial and temporal dynamics within the pre-pregnancy TDLU

**Fig. 4:**
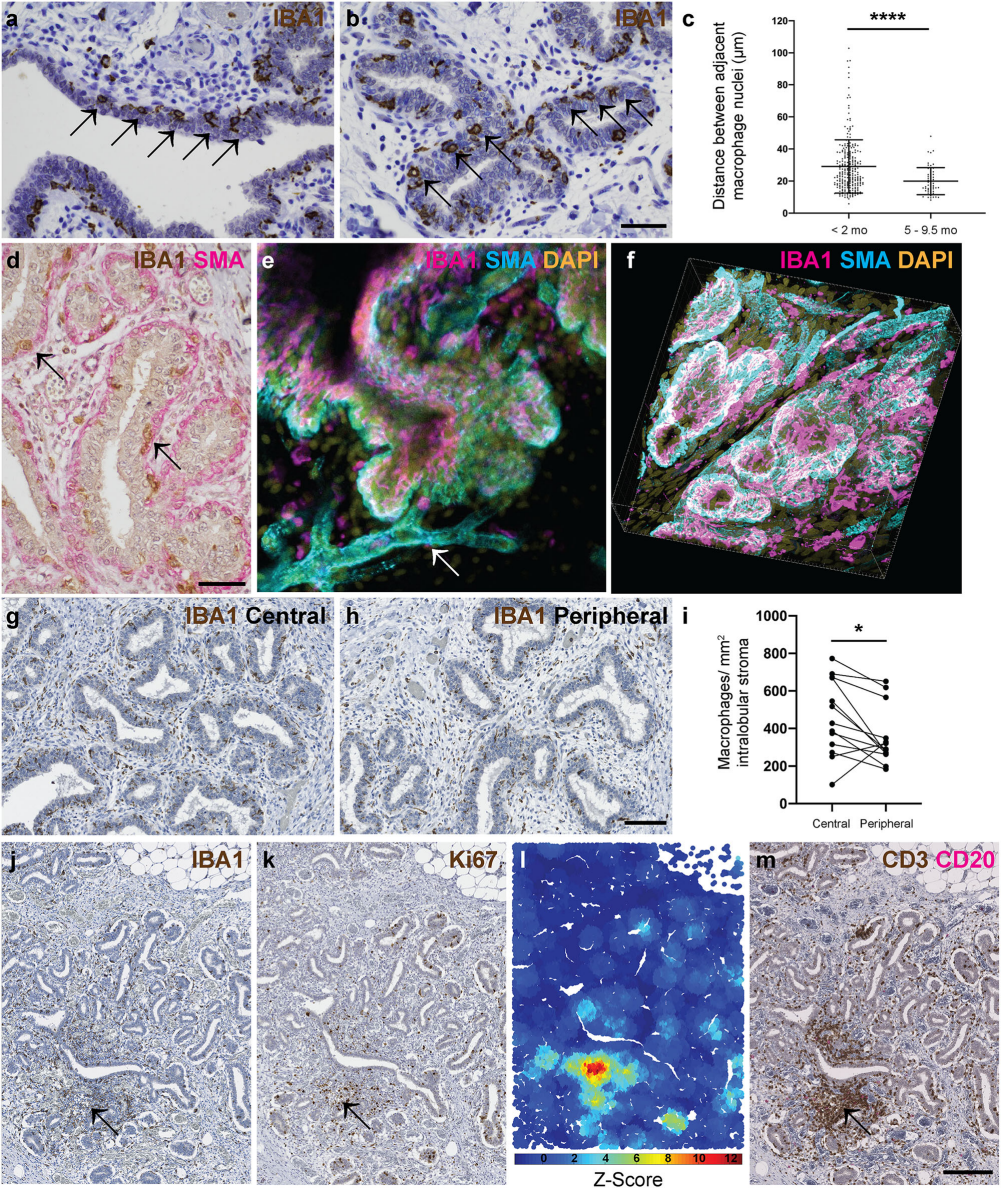
Mammary macrophages exhibit spatial and temporal dynamics. **a**, **b** IHC for IBA1 reveals macrophage periodicity (arrows) in ducts (**a**) and ductules (**b**). **c** Scatter plot demonstrating that the distributions of inter-macrophage distance differ significantly between lambs aged <2 mo and 5–9.5 mo. Dots represent inter-macrophage distances from 13 individual lambs. Bars represent mean ± standard deviation. **** *p* < 0.0001. **d** IHC for IBA1 (brown) and alpha smooth muscle actin (SMA; pink). Arrows indicate macrophages. **e**, **f** 3D confocal microscopy of optically cleared ovine mammary tissue with IF for IBA1 (magenta) SMA (cyan) and DNA (DAPI; gold). Images represent 3D maximum intensity projections. Arrow indicates blood vessel. **g**, **h** IHC for IBA1 in central (**g**) and peripheral (**h**) foci. **i** Scatter plot demonstrating significantly reduced macrophage abundance in peripheral compared to central foci. Dots represent average macrophage density for individual lambs. * *p* < 0.05. **j**–**m** Serial sections demonstrating IHC for IBA1 (**j**) Ki67 (**k**) and CD3 (brown) and CD20 (pink) (**m**) with accompanying G-O plot for Ki67 (**l**) (scale (*d*) parameter = 250 px). Arrow indicates co-localisation of stromal macrophages, a Ki67 hotspot, and a CD3^+^ lymphocyte aggregate. Images are representative of 13 (**a**, **b**, **g**, **h**), and three (**d**–**f**, **j**–**m**) biological repeats; mo, months old. All IHC images have haematoxylin counterstain. Scale bar = 40 μm (**a**, **b**, **d**); 100 μm (**g**, **h**); 200 μm (**j**, **k**, **m**).

Having established that the ovine mammary gland exhibits a distinct growth phase during pre-pubertal mammary development, we wished to compare the spatial and temporal distribution of macrophages during development pre-pregnancy. The macrophage marker ionised calcium binding adaptor molecule 1 (IBA1) is expressed by macrophages and microglia and is involved in macrophage membrane ruffling^40^. We have previously utilised this marker to detect ovine mammary macrophages^41^. In the present study, using IBA1 IHC to identify macrophages, we noted distinct periodicity of intraepithelial macrophages both within ducts and ductules (Fig. 4a, b) similar to that reported in mice^8,9^. Importantly, we identified a previously unrecognised variation in ductular macrophage density, with the distributions of inter-macrophage distance differing significantly between lambs aged less than two months old and those aged 5–9.5 months old (*P* < 0.0001) and a notably reduced inter-macrophage distance in ducts examined from the peri-pubertal animals (Fig. 4c). This increased ductular macrophage density may suggest continued accumulation over time, potentially leading to enhanced immune surveillance in animals approaching puberty, or a reorganisation of macrophage distribution following the pulse of growth associated with pre-pubertal development.

Within the developing TDLU, macrophages are intercalated within the ductal epithelial bilayer similar to the arrangement reported in the mouse^8^ (Fig. 4d–f; Supplementary Movie 1). The TDLU-associated ductal macrophages form a largely contiguous layer sandwiched between the luminal and basal epithelial cells. We hypothesise that during development pre-pregnancy this complex of macrophages is likely to fulfil an immune surveillance function, commensurate with a proposed ability to sample the epithelium through movement of cellular processes^8^ and underlining the concept of the mammary ductular microenvironment as a mucosal immune system^14^.

In addition to an abundant intraepithelial macrophage population, frequent, usually regularly spaced, macrophages are present in the ovine intralobular stroma encasing the developing TDLUs. Interestingly, these stromal macrophages are significantly more numerous in central foci than in peripheral locations (P = 0.0280) (Fig. 4g–I). This may point to stromal macrophage abundance surrounding the developing ruminant gland cistern (Fig. 1), likely reflecting an important role in immune regulation of the mammary microenvironment. However, murine stromal macrophages derived from adult mice have differing gene expression profiles compared to ductal macrophages^8^. It is thus probable that stromal macrophages also have other functions. A recent study focusing on mammary stromal macrophages has delineated a homoeostatic role for this population, with Lyve-1 expressing stromal macrophages associated with areas of hyaluronan enrichment in both mice and humans. Mice in which macrophages were depleted exhibited increased levels of hyaluronan within the stromal adipose^12^. It is therefore possible that the abundance of stromal macrophages that we have noted in the central portion of the developing ruminant TDLU may reflect mesenchymal remodelling as the gland cistern develops.

Although stromal macrophages usually exhibit a relatively regular distribution (Fig. 4g, h), we noted multifocal stromal foci in which there are more dense aggregates of IBA1 positive macrophages admixed with lymphocytes (Fig. 4j). Intriguingly, these correspond to hotspots of Ki67 expression (Fig. 4k, l). The aggregates are predominantly composed of CD3-expressing T lymphocytes, with variable numbers of CD20-expressing B lymphocytes (Fig. 4m). This prompted us to further investigate lymphocyte distribution within the developing ovine TDLUs.

### Epithelial T lymphocytes are more abundant in older lambs, and stromal lymphocytes multifocally form tertiary lymphoid structures

**Fig. 5:**
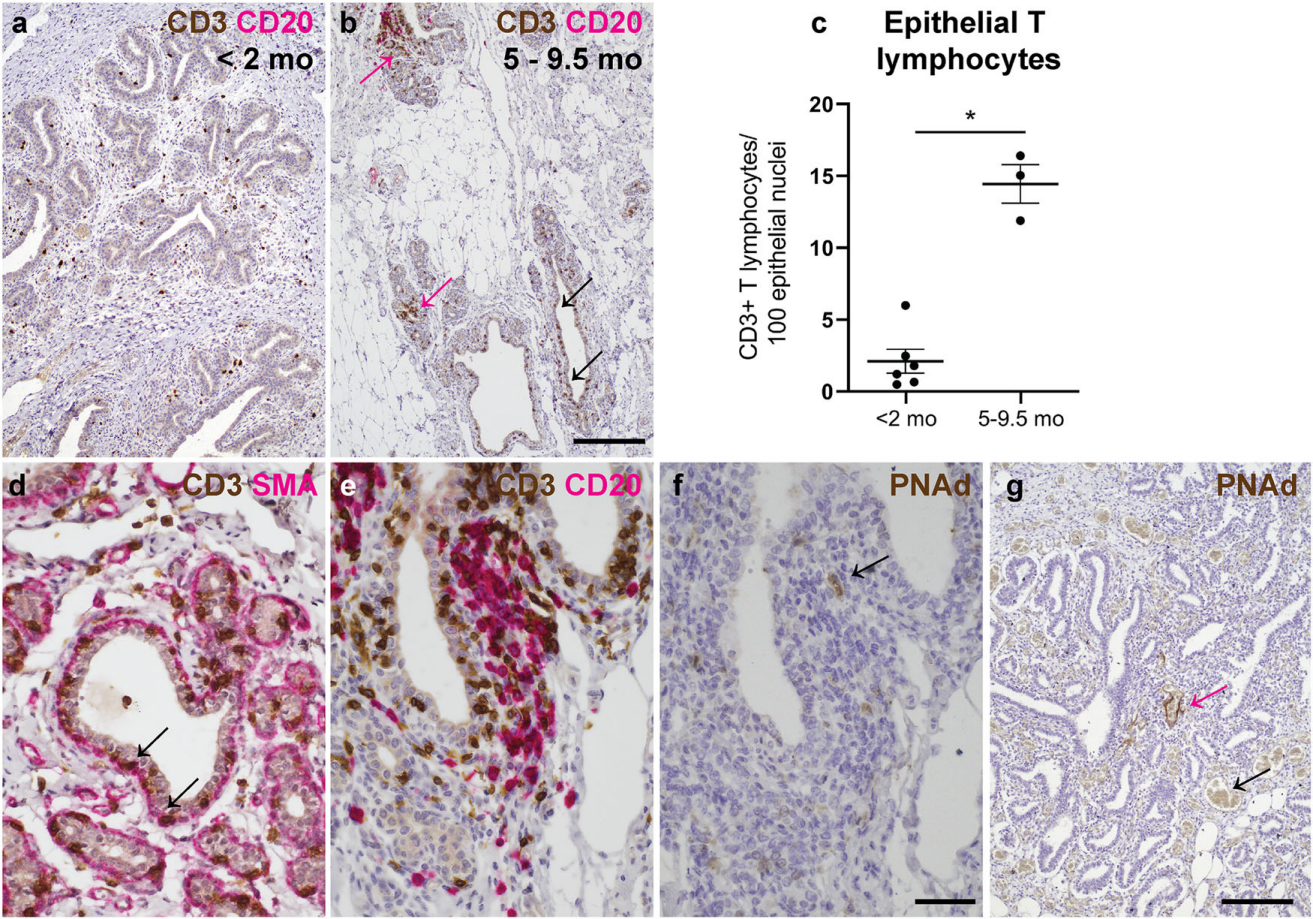
Epithelial T lymphocytes are more abundant in older lambs, and tertiary lymphoid structures are multifocally present. **a**, **b** IHC for CD3 (brown) and CD20 (pink) reveals more abundant intraepithelial (black arrows) T lymphocytes in lambs aged 5–9.5 mo. Pink arrows indicate stromal T lymphocytes. **c** Scatter plot demonstrating that the distribution of epithelial T lymphocyte counts differs significantly between younger and older lambs. Dots represent CD3^+^ lymphocyte densities from individual lambs. Bars represent mean ± standard deviation. * *p* < 0.05. **d** IHC for CD3 (brown) and SMA (pink). Arrows indicate intraepithelial lymphocytes. **e** IHC for CD3 (brown) and CD20 (pink). An aggregate of lymphocytes in a subepithelial focus exhibits a central zone of B lymphocytes surrounded by T lymphocytes. **f**, **g** IHC for PNAd. **f** Serial section of (**e**). Arrow indicates high endothelial venule within the aggregate of lymphocytes depicted in (**e**). **g** Pink arrow indicates PNAd-positive blood vessel amidst lymphocytic infiltrate. Black arrow indicates adjacent negative internal control blood vessel, demonstrating specificity of staining. Images are representative of six (**a**) and three (**b**, **d**–**g**) biological repeats; mo, months old. All IHC images have haematoxylin counterstain. Scale bar = 200 μm (**a**, **b**); 40 μm (**d**–**f**); 200 μm (**g**).

Numbers of intraepithelial CD3^+^ T lymphocytes show significantly different distributions between younger and older lambs (*P* = 0.0238) (Fig. 5a–c). CD4^+^ T helper 1 lymphocytes have previously been identified as negative regulators of mammary development and so it is tempting to speculatively associate the abundance of intraepithelial T lymphocytes in older lambs with the observed decrease in epithelial proliferation within the TDLU in this age group. The mammary immune system has been likened to a classical mucosal immune system^14^ and the presence of mammary intraepithelial lymphocytes is reminiscent of other mucosal surfaces such as the intestinal epithelium, where intraepithelial lymphocytes are common^42^. Notably, the CD3^+^ T lymphocytes in mammary intraepithelial foci frequently exhibit a similar spatial niche to intraepithelial macrophages, intercalated between the luminal and basal epithelial layers (Fig. 4d–f; Fig. 5d). We and others have previously described mammary intraepithelial lymphocytes in rabbits, mice and humans respectively^13,16,43^ and so it seems likely that this distribution is common to many species. No difference was detected in stromal CD3^+^ T lymphocyte densities between lambs in the two age groups (Supplementary Fig. 1).

Finally, we noted that some stromal aggregates of T and B lymphocytes exhibit distinct arrangement with central foci of B lymphocytes surrounded by more peripheral T lymphocytes. High endothelial venules, denoted by expression of peripheral node addressin (PNAd), are detectable within these aggregates (Fig. 5e, f) and the groupings exhibit characteristics of tertiary lymphoid structures (TLSs). TLSs are aggregates of lymphocytes possessing distinct architectural arrangement, similar to secondary lymphoid organs, which may arise in foci of chronic inflammation^44^, or secondary to autoimmune processes or neoplasia^45^. In our study, the density of TLSs did not differ significantly between neonatal and older lambs, although less tissue area per lamb was available for examination from the neonatal lambs and this may have reduced the likelihood of detecting a TLS (Supplementary Fig. 2).

The finding that TLSs are present subjacent to the mammary mucosal epithelium is particularly important given that the pre- and peri-pubertal animals studied had never lactated and never exhibited clinical evidence of mastitis. Equally, other than the presence of TLSs, there was no histological evidence of subclinical mastitis in any of the sections examined. For example, there were no neutrophilic aggregates or evidence of epithelial necrosis or architectural disruption. Indeed, mastitis would be extremely rare in this age group. That said, performing somatic cell counts of secretions, or culturing mammary tissue to assess for the presence of an intramammary infection would be required to positively assert that there were no subclinical infections present. The presence of TLSs allows inference that there is a developing competency of a local adaptive immune system being established within the parenchyma. This may reflect exposure to foreign antigens.

These observations suggest that the formation of TLSs immediately subjacent to mammary ducts in pre-pregnancy animals may constitute a hitherto unrecognised component of the mammary gland’s mucosal immune system. It seems likely that these structures may form in response to antigenic stimulation reflecting the contiguity between the mammary epithelium and the epidermis^14^. Corroborating this finding, we noted that small calibre blood vessels located in foci of mixed T and B lymphocyte aggregates, lacking the zonal organisation of TLSs, also mutifocally and selectively express endothelial PNAd (Fig. 5g). Such vascular expression of PNAd has been suggested to be associated with ‘immature’ foci in which less organised lymphocyte groupings are in the process of forming TLSs^46^. Thus the formation of TLSs is likely an active ongoing process in nulliparous lambs.

One benefit of the present study is that much larger tissue areas are typically available for analysis from ovine subjects compared to those likely available from infant breast tissue, or from normal tissue present adjacent to surgically removed breast lesions. Therefore it is possible that TLSs are a feature of the mammary mucosal immune system of other species but these structures may be rarely detectable in the samples available to researchers.

It should be noted that this study utilised ovine mammary tissue from two distinct sources, one of which comprised animals undergoing post mortem examination (Supplementary Table 1). Although animals with mammary pathology were excluded from analysis, some of the clinical subjects may have had disease in other organ systems. It is widely accepted that pathology in other body systems, and indeed various other causes of stress, may lead to elevated levels of cortisol that may impact the mammary gland during different developmental stages^47,48^.

Our work demonstrates that ovine developing mammary TDLUs have a dynamic mucosal and stromal immune microenvironment. We provide data on the growth phases and macrophage and lymphocyte fluxes occurring prior to gestation and document that TLSs are present prior to gestation and are expected to be a naturally occurring development of the mammary immune microenvironment. We also demonstrate a number of similarities between the ovine mammary gland and human breast. The lamb mammary gland thus constitutes a valuable model system that provides new insights into postnatal breast development.

## Materials and methods

### Animals

Mammary tissue was collected for this study from two separate sources. Mammary tissue was collected from female sheep aged less than one year that were submitted to the diagnostic veterinary anatomic pathology service of the Department of Veterinary Medicine, University of Cambridge (Supplementary Table 1). Additionally, mammary tissue was obtained post mortem from 2 day old–12 months old Welsh mountain ewes studied for other research purposes^49^ and euthanased under the Animals (Scientific Procedures) Act 1986. No animals exceeding 9.5 months old were included in quantitative analyses. The Ethics and Welfare Committee of the Department of Veterinary Medicine, University of Cambridge, approved the study plan relating to the use of ovine post mortem material for the study of mammary gland biology (reference: CR223). The non-regulated scientific use of post mortem mammary tissue collected from research animals was approved by the Named Veterinary Surgeon of the University of Cambridge. Together, sheep from these two sources comprised a range of breeds and crosses (Supplementary Table 1).

In all cases, macro- and microscopic post mortem examination of mammary tissue were conducted by a single American board-certified veterinary pathologist and no tissues with macro- or microscopic mammary pathology were included in the study.

### Histology

Mammary tissue was fixed in 10% neutral-buffered formalin for approximately 72 h. Tissues were processed and tissue sections were cut at 5  μm. These were stained with haematoxylin and eosin.

### Immunohistochemistry and immunofluorescence

Antibodies utilised for immunohistochemical (IHC) and immunofluorescence (IF) staining are detailed in Table 1. IHC followed a routine protocol using a PT link antigen retrieval module and high pH antigen retrieval solution (both Dako Pathology/Agilent Technologies, Stockport, UK). Primary and secondary antibodies were incubated for 1 h at room temperature. For dual IHC staining, an ImmPRESS^TM^ Duet Double Staining Polymer Kit (Vector laboratories, Peterborough, UK) was utilised. Negative control slides were prepared using isotype- and species-matched immunoglobulins or secondary antibody only.

**Table 1:** Antibodies employed for immunohistochemistry, immunofluorescence, and CUBIC.

Target	Application (IHC, immunohistochemistry; IF, immunofluorescence; CUBIC, 3D tissue clearing)	Species and clone	Dilution	Manufacturer	Catalogue number
*Primary antibodies*					
Alpha smooth muscle actin	IF; dual colour IHC	Rabbit monoclonal [EPR5368]	1:2000	Abcam	Ab124964
Alpha smooth muscle actin	CUBIC; dual colour IHC	Mouse monoclonal anti-human 1A4	1:100 (CUBIC) 1:400 (dual colour IHC)	Dako/Agilent	M0851
CD3	Dual colour IHC	Mouse monoclonal anti-human clone F7.2.38	1:250	Dako/Agilent	M7254
CD20	Dual colour IHC	Rabbit polyclonal	1:800	Thermo Fisher Scientific	RB-9013-P1
E-cadherin	Dual colour IHC	Rabbit monoclonal	1:400	Cell Signalling Technology	#3195
IBA1	IHC; dual colour IHC	Mouse monoclonal, clone 20A12.1	1:800	Millipore	MABN92
IBA1	CUBIC	Rabbit monoclonal [EPR16588]	1:400	Abcam	Ab178846
Ki67	IHC; IF	Mouse monoclonal anti-human clone MIB-1	1:100	Dako/Agilent	M7240
PNAd	IHC	Rat monoclonal anti-mouse/ human clone MECA-79	1:100	BioLegend	120802
*Secondary antibodies*					
Mouse IgG, Alexa Fluor Plus 488	IF	Goat	1:500	Thermo Fisher Scientific	A32723
Mouse IgG, Alexa Fluor 568	IF	Goat	1:500	Thermo Fisher Scientific	A11031
Rabbit IgG, Alexa Fluor Plus 488	CUBIC	Goat	1:500	Thermo Fisher Scientific	A32731
Rabbit IgG, Alexa Fluor Plus 647	CUBIC	Goat	1:500	Thermo Fisher Scientific	A32733
Rat IgG, peroxidase labelled	IHC	Goat	1:400	Vector laboratories	PI-9400

IF also followed a routine protocol. Antigen retrieval was carried out using a PT link antigen retrieval module and high pH antigen retrieval solution as detailed above. Primary antibodies were incubated overnight at 4 °C and secondary antibodies were incubated for 1 h at room temperature. Nuclei were stained with DAPI (10.9 µM) (Sigma-Aldrich/Merck Life Science UK Limited, Gillingham, UK). Slides were mounted using Vectashield^®^ Vibrance^TM^ Antifade mounting medium (catalogue H-1700; Vector laboratories, Peterborough, UK). Imaging was performed using either a Leica TCS SP8 or a Zeiss LSM780 confocal microscope.

### Tissue clearing and deep 3D imaging

Tissues were optically cleared using the CUBIC protocol^50,51^ with minor modifications as detailed below. Ovine mammary tissue was cut into slices approximately 10 mm thick and was fixed for 6–8 h in 10% neutral-buffered formalin. Tissue was then sufficiently firm to be cut into smaller pieces, on average 5 × 8 × 2 mm. Tissue pieces were subsequently immersed in CUBIC reagent 1A for 4 days at 37 °C with gentle rocking. The CUBIC reagent 1A solution was replaced daily. Samples were blocked in blocking buffer comprising normal goat serum [10% (volume per volume)] and Triton X-100 [0.5% (weight per volume)] in PBS. Samples were blocked overnight at 4 °C with gentle agitation. Tissue samples were incubated with primary antibodies (Table 1) diluted in blocking buffer for 4 days at 4 °C with gentle agitation. The samples were then washed at room temperature with gentle rocking in PBS containing Triton X-100 (0.1% (weight per weight)). Secondary antibodies (Table 1) were also prepared in blocking buffer and tissue samples were incubated in these for 2 days at 4 °C, with gentle rocking. Following thorough washing as described above, samples were incubated with DAPI (10.9 µM) (Sigma-Aldrich/Merck Life Science UK Limited, Gillingham, UK) for a minimum of 1 h at room temperature prior to further washing and immersion in CUBIC reagent 2 for at least 2 days at 37 °C with gentle rocking. Negative control tissue was prepared by omitting the primary antibody and using the secondary antibody only. Cleared and stained tissue fragments were imaged in Ibidi 35 mm glass bottom dishes (catalogue 81218-200; ibidi GmbH, Gräfelfing, Germany) using a Leica TCS SP8 confocal microscope. 3D data were visualised using ImarisViewer (Oxford Instruments, UK. Imaris Viewer: a free 3-D/4-D microscopy image viewer. https://imaris.oxinst.com/imaris-viewer Accessed 03/11/2020) and Vaa3D^52^ software.

### Slide scanning

Slides IHC stained for Ki67, IBA1, and CD3/CD20 were scanned at 40× using a NanoZoomer 2.0RS, C10730, (Hamamatsu Photonics, Hamamatsu City, Japan). Scanned sections were analysed with viewing software (NDP.view2, Hamamatsu Photonics).

### Ki67: deep learning image analysis

130 image-fields (DAB Ki67^+^ detection/haematoxylin counterstain) each covering 1.5 mm^2^ (6322 × 4581 pixels) were collected from slide scans across animals in RGB tiff format. Images were normalised across the haematoxylin/DAB colour-components using the Macenko approach^53^. Fourteen image fields were used to train the deep learning models. Firstly, a two class, semantic pixel classification network (DeepLabV3 + on a pre-trained ResNet18 backbone with output stride eight)^54,55^ was trained to provide a binary mask of ‘epithelium’ or ‘background/other’ classes. Input images were passed to the network as patches (2000/image) with dimensions 256, 256, 3 (x, y, channels) and augmented by random x/y reflection and rotation. The network was trained for 150 epochs using a batch size of eight with zero-centre normalisation under stochastic gradient descent using class-weighted cross-entropy loss. The initial learn rate was 0.001 with a drop factor every ten epochs of 0.3, a momentum of 0.9 and L2 regularisation 0.05. Patches were shuffled every epoch.

To segment Ki67^+^ and Ki67^-^ nuclei, a three-class (‘Ki67^+^ nuclei’, ‘Ki67^-^ nuclei’, ‘background/other’) Unet model^56^ was trained—again using data from fourteen, Macenko-normalised image fields. Patches (2000/image) were passed to the network with dimensions 256, 256, 3 (x, y, channels) and simple augmentation by random x/y reflection and rotation. The Unet model utilised an encoder depth of four layers with 64 filters in the first layer. The network used complete, up-convolutional expansion to yield images identically sized to the input layer. Training lasted for fifty epochs, using batch size of eight with zero-centre normalisation under stochastic gradient descent utilising cross-entropy loss. The initial learn rate was 0.05, dropping every ten epochs by 0.1 under momentum 0.9 and L2 regularisation 0.0001.

Models were trained using MATLAB R2020 and the Deep Learning Toolbox. The trained models, test data alongside all training hyper-parameters and final layer-weightings are available for download at BioStudies database (http://www.ebi.ac.uk/biostudies) under accession number S-BSST528. Both models were tested against entirely unseen data (the other 116 fields) and the results validated using boundary overlays and manual image review by an American board-certified veterinary pathologist. The ratio (pixel area) of Ki67^+^ to Ki67^-^ nuclei in the epithelium of each image field was calculated using the epithelial segmentation mask from the DeepLabV3 + ResNet18 model to mask the Unet segmentations for each nuclear phenotype.

### Ki67: Getis-Ord spatial analyses

Per-nuclei intensity and spatial location data were extracted using CellProfiler^57^ as described in the previous work^58^. Statistically significant, spatial ‘congregations’ of Ki67^+^ nuclei relative to what would be expected by random chance were identified using the Getis-Ord GI* statistical approach^59^. Ki67^+^ and Ki67^-^ nuclear objects segmented by the Unet model were used to define the centroid position for both nuclear phenotypes in an image-field. The spatial concentration of values *x*_*j*_ for *j* values within a distance *d* of the value *x*_*i*_ were then defined. To do this, the ratio ${G}_{i}^{\ast }$ was defined as

1 $${G}_{i}^{\ast }(d)=\frac{{\sum }_{j=1}^{n}{{w}_{{ij}}(d)x}_{j}}{{\sum }_{j=1}^{n}{x}_{j}}$$

here, *w*_*ij*_*(d)* defines the numerator contribution of the ratio depending on the distance *d*. For example, using *w*_*ij*_*(d)* = 1, if *d*_*ij* _< *d* else; *w*_*ij*_*(d)* = 0 if *d*_*ij*_ > *d*. From here, the Getis-Ord statistic is given by

2 $$Z\left[{G}_{i}^{\ast }(d)\right]=\frac{[{G}_{i}^{\ast }(d)-E({G}_{i}^{\ast }(d))]}{\sqrt{{{{{{{\rm{var}}}}}}}\,{G}_{i}^{\ast }(d)}}$$

where $E({G}_{i}^{\ast}(d))$ represents the expected fraction of items within *d*, assuming a completely random distribution calculated as

3 $$E\left({G}_{i}^{\ast }(d)\right)=\frac{\mathop{\sum}\nolimits_{j}{\omega }_{{ij}}(d)}{n-1}$$

The value $Z\left[{G}_{i}^{\ast }(d)\right]$ now describes the difference in the fraction of values within the distance *d* from location *i* from what would be expected by random chance relative to the standard deviation. Here, we discretise each image field into a grid and value *x*_*i*_ is defined as the number of nuclei of a certain phenotype in the grid position *i*^58^.

### Assessment of macrophage periodicity

Macrophage periodicity was defined on IBA1 IHC stained sections as a segment of at least 4 evenly spaced intraepithelial macrophages. Spacing between macrophages was measured from the central aspect of the macrophage nucleus to the central aspect of the next macrophage nucleus using the NDP.view2 software. The centre of the cell was inferred in instances where the nucleus was not perfectly sectioned but where there was a strong impression of the nuclear position. Measurements were made parallel to the epithelium. Groups of macrophages were excluded unless they constituted a very tightly clustered small group of less than 3 macrophages in a region of clear periodicity.

### Sampling for stromal macrophage and T lymphocyte counts

Using NDP.view2 slide viewing software, eight count boxes (400 × 230 µm; 4 per central or peripheral location for macrophages) were placed per slide, separately for macrophage and T lymphocyte quantification, at ×1.3 magnification where only ductal structure, but not staining, was discernible, to prevent placement bias while maximising the epithelium sampled. Boxes in any fields with slide cutting artefacts or scanning focus artefacts were repositioned. For the macrophage analysis, selected fields were classified as ‘peripheral’ if sampling the edge of ductal/lobular epithelial structures, advancing into surrounding adipose tissue, or ‘central’ if the sampled mammary parenchyma was entirely surrounded by other mammary epithelial units.

### Cell quantification for stromal macrophage and T lymphocyte counts

Cells with >50% of their nucleus within the count box, or if equivocal, those along the top and right edges, were counted. A macrophage was counted as an area of IBA-1 expression that was at least 50% of the average luminal epithelial cell nucleus in that count box. ‘Stromal macrophage’ count was normalised to intralobular stromal area, determined using the NDP.view2 freehand annotation tool. ‘Epithelial T lymphocytes’ had >50% of their cytoplasmic perimeter contacting the basement membrane, with counts normalised per 100 luminal epithelial nuclei in the count box. ‘Stromal T lymphocyte’ count was normalised to total stromal area within the count box, determined using the NDP.view2 freehand annotation tool.

### Lymphocyte aggregate qualitative description and density

TLSs were defined as a discrete B lymphocyte aggregates with a distinct adjacent T lymphocyte area following previously published work^60^. TLS were counted by two independent observers (DN and KH). Where there was a discrepancy between the counts made by the two investigators, count results from both investigators were reviewed and the final decision on count was made by the American board-certified veterinary pathologist having reviewed the identified structures. The area of mammary tissue analysed for each lamb was determined as above, using the NDP.view2 freehand annotation tool.

### Statistics and reproducibility

Data were recorded using Excel (Supplementary Data 1) and analysed with GraphPad Prism 8.4.3. Numerical data were assessed for normality using the D’Agostino normality test. Statistical significance was then assessed using the Mann Witney test for non-parametric data, with the exception of Fig. 4i where a paired two-tailed t-test was used.

### Reporting summary

Further information on research design is available in the Nature Research Reporting Summary linked to this article.

## Supplementary information

Supplementary Information

Description of Supplementary Files

Supplementary Movie 1

Supplementary Data 1

Reporting Summary

## Data Availability

Example image data are available for download at the BioStudies database (http://www.ebi.ac.uk/biostudies) under accession number S-BSST528. The source data for the graphs and charts in the figures is available as Supplementary Data 1 and any remaining information is obtained from the corresponding authors upon reasonable request.

## Code availability

Code (MATLAB R2020), trained deep learning models and test data including full details of training hyperparameters and final layer weightings are available for download at BioStudies database (http://www.ebi.ac.uk/biostudies) under accession number S-BSST528.
