# Supplementary material for: Developing ovine mammary terminal duct lobular units have a dynamic mucosal and stromal immune microenvironment
Source: Commun Biol. 2021 Aug 20;4:993. doi: 10.1038/s42003-021-02502-6 (PMC8379191; doi:10.1038/s42003-021-02502-6)
Supplement: Supplementary file 1 — Supplementary Information [file 42003_2021_2502_MOESM1_ESM.pdf]

Developing ovine mammary terminal duct lobular units have a dynamic mucosal and stromal immune microenvironment

Supplementary information

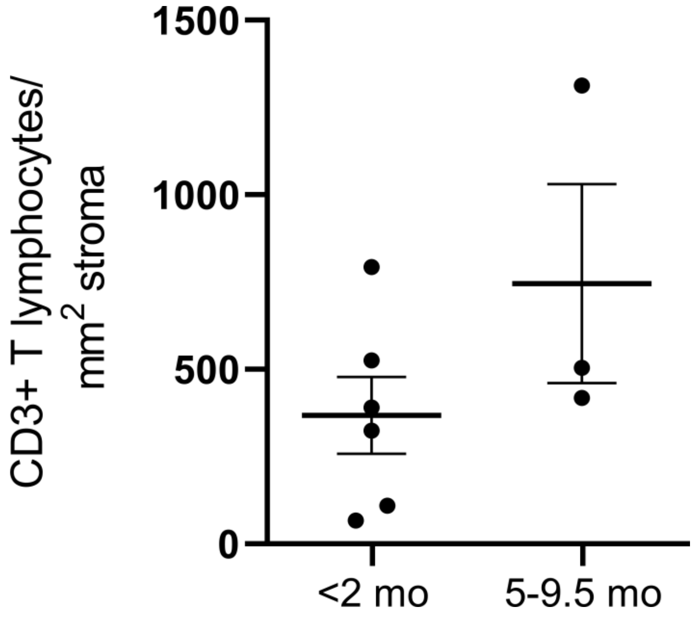

Supplementary Figure 1. The overall abundance of stromal T lymphocytes is similar in younger and older lambs. Scatter plot demonstrating distributions of stromal T lymphocytes in lambs less than two months old (< 2 mo) and aged 5-9.5 months (5-9.5 mo). Dots represent CD3+ lymphocyte densities from individual lambs. Bars represent mean +/- standard deviation.

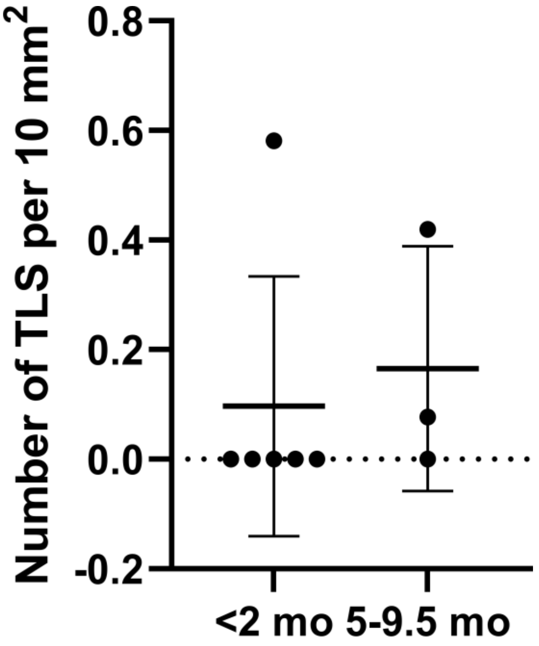

Supplementary Figure 2. The density of tertiary lymphoid structures (TLS) does not differ significantly between neonatal and older lambs. Scatter plot demonstrating density of TLS in lambs less than two months old (< 2 mo) and aged 5-9.5 months (5-9.5 mo). Dots represent individual lambs. Bars represent mean +/- standard deviation.

Supplementary Table 1. Data for individual sheep used in the study.
do, days old; wo, weeks old; mo, months old; SUP, supplementary figure.

Case number	Source	Age	Breed	UK meteorological season died/ euthanased	Figures for which used
1	Diagnostic PM	1 do	Lleyn cross	Winter	2; 4
2	Diagnostic PM	2 do	Lleyn cross	Winter	1; 4; 5; SUP1; SUP2
3	Diagnostic PM	1 do	Lleyn cross	Winter	1; 4
4	Diagnostic PM	1 do	Lleyn cross	Winter	1; 2; 4; 5; SUP1; SUP2
5	Diagnostic PM	1 do	Lleyn cross	Winter	2; 4
6	Diagnostic PM	2 wo	Lleyn cross	Winter	4
7	Diagnostic PM	2 wo	Lleyn cross	Winter	2; 4
8	Diagnostic PM	1 do	Lleyn cross	Winter	2; 3
9	Diagnostic PM	1 do	Lleyn cross	Winter	2
10	Diagnostic PM	1 do	Lleyn cross	Winter	2; 5; SUP1; SUP2
11	Diagnostic PM	1 do	Mule cross	Winter	5; SUP1; SUP2
12	Diagnostic PM	1 mo	Suffolk cross	Spring	2
13	Diagnostic PM	2 mo	Suffolk cross	Summer	2; 4
14	Diagnostic PM	2 mo	Lleyn	Spring	1; 4; 5; SUP1; SUP2
15	Diagnostic PM	1 do	Mule cross	Winter	2; 5; SUP1; SUP2
16	Diagnostic PM	7 mo	Not recorded	Autumn	2; 3; 4; 5; SUP1; SUP2
17	Diagnostic PM	5 mo	Texel cross	Summer	1; 2; 3; 4; 5; SUP1; SUP2
18	Diagnostic PM	6 mo	Lleyn	Summer	4
19	Diagnostic PM	6 mo	Lleyn	Summer	4
20	Research	8 mo	Welsh mountain	Winter	2; 4; 5; SUP1; SUP2
21	Research	9.5 mo	Welsh mountain	Spring	2
22	Research	9.5 mo	Welsh mountain	Winter	2
23	Research	11 mo	Welsh mountain	Winter	4
24	Research	12 mo	Welsh mountain	Summer	4
25	Research	11 mo	Welsh mountain	Summer	4; 5
