# Supplementary material for: Developing ovine mammary terminal duct lobular units have a dynamic mucosal and stromal immune microenvironment
Source: Commun Biol. 2021 Aug 20;4:993. doi: 10.1038/s42003-021-02502-6 (PMC8379191; doi:10.1038/s42003-021-02502-6)
Supplement: Supplementary file 2 — Description of Supplementary Files [file 42003_2021_2502_MOESM2_ESM.pdf]

Description of Additional Supplementary Files

File name: Supplementary Movie 1

Description: Three-dimensional rendering demonstrating the intimate association between myoepithelial cells (SMA; grey) and macrophages (IBA1; magenta) in CUBIC-cleared developing lamb mammary TDLUs.

File name: Supplementary Data 1

Description: Source data for graphs and charts.
