# Supplementary material for: Developing ovine mammary terminal duct lobular units have a dynamic mucosal and stromal immune microenvironment
Source: Commun Biol. 2021 Aug 20;4:993. doi: 10.1038/s42003-021-02502-6 (PMC8379191; doi:10.1038/s42003-021-02502-6)
Supplement: Supplementary file 5 — Reporting Summary [file 42003_2021_2502_MOESM5_ESM.pdf]

nature research

Corresponding author(s): John Wills and Katherine Hughes

Last updated by author(s): Jun 18, 2021

Reporting Summary

Nature Research wishes to improve the reproducibility of the work that we publish. This form provides structure for consistency and transparency in reporting. For further information on Nature Research policies, see our Editorial Policies and the Editorial Policy Checklist.

Statistics

For all statistical analyses, confirm that the following items are present in the figure legend, table legend, main text, or Methods section.

n/a	Confirmed	
☐	☒	The exact sample size (n) for each experimental group/condition, given as a discrete number and unit of measurement
☐	☒	A statement on whether measurements were taken from distinct samples or whether the same sample was measured repeatedly
☐	☒	The statistical test(s) used AND whether they are one- or two-sided<br>*Only common tests should be described solely by name; describe more complex techniques in the Methods section.*
☒	☐	A description of all covariates tested
☐	☒	A description of any assumptions or corrections, such as tests of normality and adjustment for multiple comparisons
☒	☐	A full description of the statistical parameters including central tendency (e.g. means) or other basic estimates (e.g. regression coefficient) AND variation (e.g. standard deviation) or associated estimates of uncertainty (e.g. confidence intervals)
☒	☐	For null hypothesis testing, the test statistic (e.g. F, t, r) with confidence intervals, effect sizes, degrees of freedom and P value noted<br>*Give P values as exact values whenever suitable.*
☒	☐	For Bayesian analysis, information on the choice of priors and Markov chain Monte Carlo settings
☒	☐	For hierarchical and complex designs, identification of the appropriate level for tests and full reporting of outcomes
☒	☐	Estimates of effect sizes (e.g. Cohen's d, Pearson's r), indicating how they were calculated

Our web collection on statistics for biologists contains articles on many of the points above.

Software and code

Policy information about availability of computer code

Data collection	DeepLabV3+ on a pre-trained ResNet18 backbone with output stride eight.<br>Unet model.<br>Refs: He, K., Zhang, X., Ren, S. & Sun, J. in 2016 IEEE Conference on Computer Vision and Pattern Recognition (CVPR). 770-778.<br>Chen, L.-C., Zhu, Y., Papandreou, G., Schroff, F. & Adam, H. 833-851 (Springer International Publishing).<br>Ronneberger, O., Fischer, P. & Brox, T. 234-241 (Springer International Publishing).
Data analysis	MATLAB R2020 and the Deep Learning Toolbox

For manuscripts utilizing custom algorithms or software that are central to the research but not yet described in published literature, software must be made available to editors and reviewers. We strongly encourage code deposition in a community repository (e.g. GitHub). See the Nature Research guidelines for submitting code & software for further information.

Data

Policy information about availability of data

All manuscripts must include a data availability statement. This statement should provide the following information, where applicable:

- Accession codes, unique identifiers, or web links for publicly available datasets
- A list of figures that have associated raw data
- A description of any restrictions on data availability

Trained deep learning models, test data alongside all training hyper-parameters, and final layer-weightings are available for download at BioStudies database (http://www.ebi.ac.uk/biostudies) under accession number S-BSST528.

Field-specific reporting

Please select the one below that is the best fit for your research. If you are not sure, read the appropriate sections before making your selection.

☒ Life sciences ☐ Behavioural & social sciences ☐ Ecological, evolutionary & environmental sciences

For a reference copy of the document with all sections, see nature.com/documents/nr-reporting-summary-flat.pdf

Life sciences study design

All studies must disclose on these points even when the disclosure is negative.

Sample size	For the Ki67 analyses, we assumed a standard deviation of 8 units, and using an online tool, determined that the study would require a sample size of 4 for the test group and 8 for the reference group (as we had more samples available from younger lambs) to achieve a power of 80% and a level of significance of 5% (two sided), for detecting our estimated true difference in means between the test and the reference group of 15 units. Reference: Dhand, N. K., & Khatkar, M. S. (2014). Statulator: An online statistical calculator. Sample Size Calculator for Comparing Two Independent Means. http://statulator.com/SampleSize/ss2M.html
Data exclusions	One research animal was excluded because it had received cortisol administration in utero.
Replication	Experimental findings were based on biological repeats between animals rather than experimental repeats, with the exception of multiple measurements taken from 13 animals (13 biological repeats) for macrophage periodicity analysis.
Randomization	Animals were allocated into experimental groups based on age.
Blinding	For deep learning image analysis, investigators were blind to the age of the animal. For export of images and placement of count boxes, selection of tissue area was made at 1.3x magnification where only ductal structure, but not staining, was discernible, to prevent placement bias.

Reporting for specific materials, systems and methods

We require information from authors about some types of materials, experimental systems and methods used in many studies. Here, indicate whether each material, system or method listed is relevant to your study. If you are not sure if a list item applies to your research, read the appropriate section before selecting a response.

Materials & experimental systems

n/a	Involved in the study	
☐	☒	Antibodies
☒	☐	Eukaryotic cell lines
☒	☐	Palaeontology and archaeology
☐	☒	Animals and other organisms
☒	☐	Human research participants
☒	☐	Clinical data
☒	☐	Dual use research of concern

Methods

n/a	Involved in the study	
☒	☐	ChIP-seq
☒	☐	Flow cytometry
☒	☐	MRI-based neuroimaging

Antibodies

Antibodies used	Provided in Table 1.
Validation	Manufacturers extremely rarely validate commercial antibodies against ovine epitopes. The measures taken to validate each antibody for this study are described below.<br><br>Alpha smooth muscle actin: Validated by an American Board-Certified veterinary pathologist using appropriate internal positive control tissues on the tissue sections (e.g. smooth muscle in the wall of blood vessels). Negative internal control tissues assessed. One of the alpha smooth muscle actin antibodies used (MO851) has been previously used in a peer-reviewed publication describing expression of alpha smooth muscle actin in sheep tissues (ref below).<br><br>CD3: Validated by an American Board-Certified veterinary pathologist using appropriate positive control tissue (lymph node). Negative internal control tissues assessed. Antibody has been previously used in a peer-reviewed publication describing expression of CD3 in sheep tissues (ref below).<br><br>CD20: Validated by an American Board-Certified veterinary pathologist using appropriate positive control tissue (lymph node). Negative internal control tissues assessed.<br><br>E-cadherin: Validated by an American Board-Certified veterinary pathologist using appropriate internal positive control tissues on the

tissue sections (e.g. epidermis overlying mammary glands; sebaceous glands etc). Negative internal control tissues assessed. Antibody has been previously used in a peer-reviewed publication describing expression of E-cadherin in sheep tissues (ref below).

IBA1: Validated by an American Board-Certified veterinary pathologist using appropriate positive control tissue (lymph node sinuses exhibiting sinus histiocytosis). Negative internal control tissues assessed. One of the IBA1 antibodies used (MABN92) has been previously used in a peer-reviewed publication describing expression of IBA1 in sheep tissues (ref below).

Ki67: Validated by an American Board-Certified veterinary pathologist using appropriate positive control tissue (lymph node with follicular proliferation). Negative internal control tissues assessed.

PNAd: Validated by an American Board-Certified veterinary pathologist using appropriate positive control tissue (lymph node). Negative internal control tissues assessed.

Ref: Hardwick, L. J. A., Phythian, C. J., Fowden, A. L. & Hughes, K. Size of supernumerary teats in sheep correlates with complexity of the anatomy and microenvironment. J Anat 236, 954-962, doi:10.1111/joa.13149 (2020).

Animals and other organisms

Policy information about studies involving animals; ARRIVE guidelines recommended for reporting animal research

Laboratory animals	Female Welsh mountain ewes 2 day old – 12 months old.
Wild animals	The study did not involve wild animals.
Field-collected samples	Mammary tissue was collected from female sheep aged less than one year that were submitted to the diagnostic veterinary anatomic pathology service of the Department of Veterinary Medicine, University of Cambridge.
Ethics oversight	The Ethics and Welfare Committee of the Department of Veterinary Medicine, University of Cambridge, approved the study plan relating to the use of ovine post mortem material for the study of mammary gland biology (reference: CR223). The non-regulated scientific use of post mortem mammary tissue collected from research animals was approved by the Named Veterinary Surgeon of the University of Cambridge.

Note that full information on the approval of the study protocol must also be provided in the manuscript.
